# Rad51 supports triple negative breast cancer metastasis

**DOI:** 10.18632/oncotarget.1923

**Published:** 2014-04-27

**Authors:** Adrian P Wiegmans, Fares Al-Ejeh, Nicole Chee, Pei-Yi Yap, Julia J Gorski, Leonard Da Silva, Emma Bolderson, Georgia Chenevix-Trench, Robin Anderson, Peter T Simpson, Sunil R Lakhani, Kum Kum Khanna

**Affiliations:** ^1^ QIMR Berghofer Medical Research Institute, Signal Transduction Laboratory, Herston Rd, Herston QLD 4006, Australia; ^2^ Queens University Belfast, Dentistry and Biomedical Science, Lisburn Rd, Belfast, BT5 7BL, UK; ^3^ The University of Queensland, UQ Centre for Clinical Research, Herston, Brisbane, QLD 4006, Australia; ^4^ The University of Queensland, School of Medicine, Herston, Brisbane, QLD 4006, Australia; ^5^ The University of Queensland, Institute of Health and Biomedical Innovation, TRI, Woolloongabba, Brisbane, QLD 4102, Australia; ^6^ Cancer Genetics Laboratory, Queensland Institute of Medical Research, Herston Rd, Herston QLD 4006, Australia; ^7^ Metastasis Research Laboratory, Peter MacCallum Cancer Centre, St Andrews Place, East Melbourne Vic 3002, Australia; ^8^ Department of Oncology, Sir Peter MacCallum Cancer Centre, The University of Melbourne, Parkville Vic 3052, Australia; ^9^ Pathology Queensland: The Royal Brisbane & Women's Hospital, Brisbane, Herston QLD 4006, Australia

**Keywords:** RAD51, metastasis, breast cancer, DNA damage, c/EBPbeta, metastatic cancer

## Abstract

In contrast to extensive studies on familial breast cancer, it is currently unclear whether defects in DNA double strand break (DSB) repair genes play a role in sporadic breast cancer development and progression. We performed analysis of immunohistochemistry in an independent cohort of 235 were sporadic breast tumours. This analysis suggested that RAD51 expression is increased during breast cancer progression and metastasis and an oncogenic role for RAD51 when deregulated. Subsequent knockdown of RAD51 repressed cancer cell migration in vitro and reduced primary tumor growth in a syngeneic mouse model in vivo. Loss of RAD51 also inhibited associated metastasis not only in syngeneic mice but human xenografts and changed the metastatic gene expression profile of cancer cells, consistent with inhibition of distant metastasis. This demonstrates for the first time a new function of RAD51 that may underlie the proclivity of patients with RAD51 overexpression to develop distant metastasis. RAD51 is a potential biomarker and attractive drug target for metastatic triple negative breast cancer, with the capability to extend the survival of patients, which is less than 6 months.

## INTRODUCTION

The molecular transformation of normal breast epithelia to tumor requires the accumulation of a number of mutations in key metabolic pathways. This is most readily achieved by deregulation of cellular DNA repair pathways, resulting in increased genomic instability. The aggressive triple negative breast cancer (TNBC) subtype lacks expression of hormone receptors (ER, PR and HER2) and therefore progression is determined by specific molecu-lar aberrations critically important for cell survival. We hypothesised that aberrant DNA damage response (DDR) may drive and contribute to metastatic progression of sporadic TNBC.

Much of the evidence linking defective DNA damage response (DDR) with breast cancer susceptibility comes through studies of familial breast cancer. Many of the breast cancer predisposition genes identified that mediate homologous recombination (HR) repair of DNA damage, including *BRCA1* and *BRCA2*, are mutated or epigenetically silenced [1, 2]. However, no inactivating mutations of RAD51, a crucial HR protein, have been reported in tumours. Paradoxically, Rad51 is overexpressed in multiple tumor types, including breast cancer [3]. Overexpression of RAD51 is due to increased transcription, reduced methylation and/or stabilization of the protein but not due to amplification of the *RAD51* gene [4, 5]. Overexpression of RAD51 has also been suggested as a driver of aberrant recombination, resulting in excess DNA damage in precancerous cells. These drive pathological recombination events such as chromosomal amplifications, deletions and translocations resulting in loss of heterozygosity and aneuploidy. These events can lead to cancer development and progression to metastasis [6, 7]. Cells that overexpress RAD51 exhibit disruption of cell cycle, resistance to apoptotic signals and associated resistance to DNA damaging agents (radiotherapy) and chemotherapy [8-10]. Hence depletion of RAD51 by siRNA and shRNA results in potential sensitization to various agents as seen in radiation treatment of pancreatic cancer and multiple myeloma [11], and in combination therapies for non small cell lung cancer (NSCLC) with gemcitabine [12] and glioma with temozolomide [13]. However, despite these studies, the molecular mechanisms of RAD51-mediated cancer progression have not been fully elucidated.

We utilized *in silico* analysis of gene expression datasets, clinical pathology and cellular biology studies to show that one of the DNA repair genes *RAD51* was over represented in high grade and metastatic breast carcinomas. We also utilized animal models to show that RAD51 is required for metastasis and discovered that RAD51 is a potential transcriptional co-factor of c/EBPβ and supports metastatic expansion of cancer cells via regulating changes in gene expression. These are important discoveries for the development of new therapeutic regimes and for successful treatment of aggressive breast cancer, in particular metastases.

## RESULTS

### RAD51 expression correlates with high-grade metastatic breast tumors and poor prognosis

**Figure 1:**
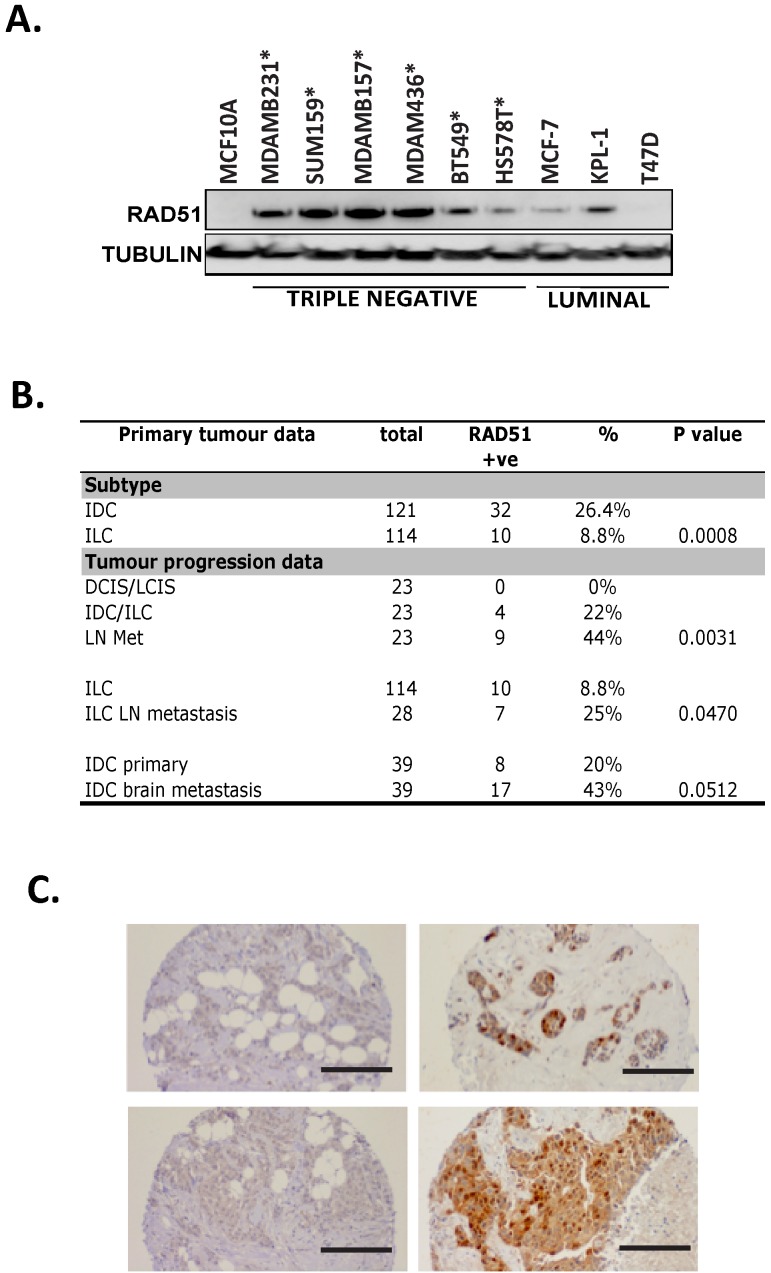
High RAD51 expression is observed in basal breast cell lines and metastatic patient samples (A) Ten breast cancer cell lines were analysed for RAD51 protein expression. Triple negative breast cancer lines are marked with an asterix. (B) 235 primary breast cancers were analysed by tissue microarray for RAD51 expression. P vales were obtained using Chi squared test with Yates correction (95% confidence interval) and Fisher's exact test to compare RAD51 status between defined subgroups of samples. (C) Histological comparison of RAD51 expression shows high levels in metastatic lymph node tissue samples (right panels) compared to matched primary breast tumor (left panels −100X magnification, bar 50µm).

Increased RAD51 expression has been correlated with poor clinical outcome in lung cancer, prostate cancer and in ER+ breast cancer [14-17]. Comparison of breast cancer cell lines revealed that RAD51 protein expression was at two fold or higher in 9 of 12 (75%) triple negative cell lines, 1 of 5 (20%) luminal and 2 of 3 (67%) HER2+ breast cancer cell lines when compared to the MCF10A cell line (near normal mammary epithelial cell line derived from a patient with benign fibrosarcoma)(Data not shown). An example of the difference in expression in triple negative breast cancer cell lines versus luminal is shown in Figure 1A. Of note we found RAD51 expression levels to be independent of both proliferation rate of the cells (R^2^=0.0012, confirmed by contrasting RAD51 expression to S-phase regulated genes PCNA /Geminin) and RAD51 functional status with varied formation of foci in response to irradiation (Supplementary Figure 1A-C). To validate our cell line data, we proceeded to evaluate RAD51 expression by immunohistochemistry in an independent cohort of 235 were sporadic tumours (Figure 1B). We found overall that 42/235 (17.8%) were positive for nuclear RAD51. In the ILC cases with lymph nodes metastases (LN mets), we found a significant enrichment of nuclear RAD51 in metastatic compared to the primary tumors (p=0.047, Figure 1B). Moreover, in 23 cases of matched in situ carcinoma, invasive carcinoma and LN metastases from the same patients, RAD51 levels increased with higher stage lesions (p=0.003, Figure 1B). We also investigated RAD51 expression in a series of matched primary IDC and brain metastasis from the same patients (n=39). Interestingly we observed a higher frequency of positivity in these distant metastases (17/39, 43%) compared to the primary tumors (8/39, 20%), which approached significance (p=0.0512, Figure 1C). We next investigated *RAD51* mRNA expression and correlation with histopathological parameters in the METABRIC (Molecular Taxonomy of Breast Cancer International Consortium) dataset [18]. Fifty two percent of tumours had high *RAD51* mRNA expression, which was significantly associated with aggressive clinicopathological features (Supplementary Table 1), including diagnosis at younger age, high histological grade, high proliferation index, HER2-overexpression, absence of hormonal receptors (ER/PR), presence of basal like phenotypes and triple-negative phenotypes (Supplementary Table 1). RAD51 expression was higher in tumors with TP53 mutation and correlated with worse overall survival at 3 and 5 years (Supplementary Table 1). Overall in silico revealed similar RAD51 expression trends to those observed with cell lines and patient immunohistochemistry, consistent with the notion that RAD51 expression is increased during breast cancer progression and metastasis, suggesting an oncogenic role when deregulated. Consistent with previous work [3] our observations demonstrate RAD51 is more highly expressed in aggressive breast cancer when compared to normal tissue.

### RAD51 knockdown affects metastasis in a syngeneic tumor model *in vivo*

**Figure 2:**
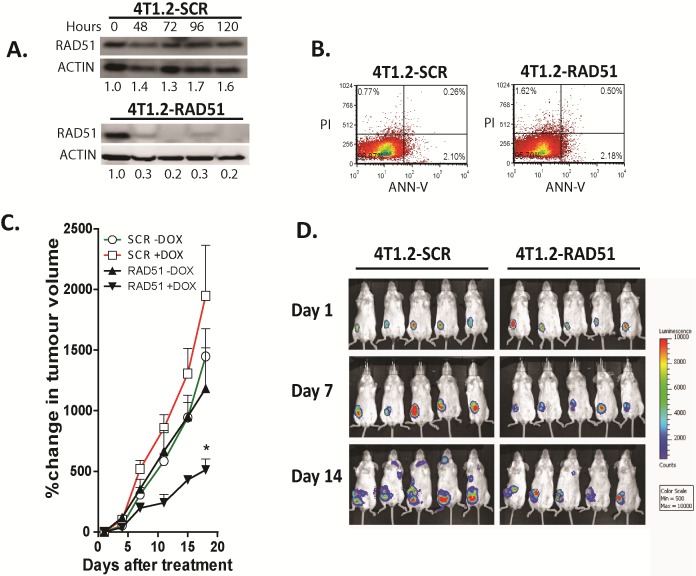
Depletion of RAD51 inhibits tumor growth and metastatic progression in a syngeneic murine breast cancer model (A) Knockdown of RAD51 protein expression was achieved using an inducible RAD51mir construct after 48 hours exposure to 5µg/ml Doxycycline. (B) Viability of 4T1.2 cells induced with doxycycline for 7 days with and without depletion of RAD51 were analysed by propidium iodide uptake. (C) Depletion of RAD51 was induced in cohorts of 10, 5 week-old female Balb/C mice with 5µg/ml Doxycycline after establishing 40mm^3^ tumors from 1×10^6^ cells of 4T1.2-RAD51mir and 4T1.2-SCRmir injected into the mammary fat pad. Cohorts were monitored for tumor growth over 18 days, with uninduced mice serving as controls *p=0.0013 +SD. (D) Primary tumor and metastatic burden was also followed by luciferase expression.

**Figure 3:**
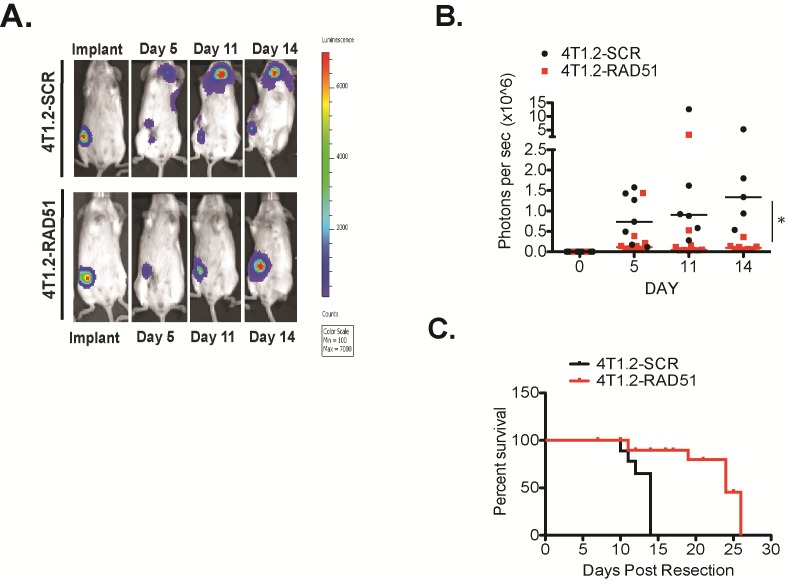
RAD51 supports metastatic burden in vivo Cohorts of 10, 5 week-old female Balb/C were induced with 5µg/ml Doxycycline after establishing 40mm3 tumors from 1×10^6^ cells of 4T1.2-RAD51mir and 4T1.2-SCRmir injected into the mammary fat pad. Primary tumours were resected and tumour burden followed for 2 weeks. (A) Tumours competent for RAD51 spontaneosuly metastasized, while tumours depleted of RAD51 only re-established at the primary site. (B) Mice with tumours competent for RAD51 displayed higher median tumour burden (black) compared to mice harbouring tumours depleted of RAD51 (red) as measured by total luminescence *p=0.024 +/−SEM. (C) The RAD51 knockdown cohort displayed a significant survival advantage (red line) compared to the control RAD51 competent cohort (black line) (p=0.025) +/−SEM.

Next, we investigated the functional contribution of RAD51 to primary tumour growth and metastasis formation in vivo. using 4T1.2 cells, (a syngeneic mouse mammary tumour model of spontaneous metastasis). We engineered the 4T1.2 lines to express a doxycycline regulated shRNA targeting Rad51. The pooled transfectants showed reduced RAD51 protein expression by 70-80% (Fig 2A), with no change in cell growth with RAD51 depleted cells doubling rate 32.3 hours versus SCR control cells 28.6 hours (p=0.067) and viability compared to cells transfected with a scrambled shRNA as a control (Fig 2B). Both cell lines expressing the firefly luciferase gene were orthotopically implanted into mammary fat pads of immunocompetent mice to determine if RAD51 knockdown impacts on primary tumour growth and/or metastasis. The depletion of RAD51 expression in 4T1.2 cells lead to a significant delay in primary tumor growth in vivo (p=0.0013)(Fig 2C), with sustained depletion of RAD51 at the primary site and overexpression observed in metastatic tumours (Supplementary Figure 2). Luciferase levels representing the presence of tumour cells in distant organs were decreased in mice transplanted with shRAD51 4T1.2 cells as compared to shControl 4T1.2 cells (SCR) at day 14 (Fig 2D), even when the luciferase activities were normalized to the decreased primary tumor size observed with shRAD51 4T1.2 cells. We hypothesize that the observed delay in metastasis was due to poor seeding and growth of the RAD51 depleted cancer cells at secondary sites. To analyse whether RAD51 is required to support metastatic cell growth we repeated the above experiment after resection of same size primary tumours. As expected from the above data, depletion of RAD51 expression in the absence of the primary tumour inhibited onset of metastatic lesions. After 14 days RAD51 depletion resulted in regrowth of tumor at the original primary site compared with control mice that displayed disseminated metastatic tumours to the bone and brain (Figure 3A). Overall tumour burden median was lower in the RAD51 depleted cohort compared to the control cohort (Figure 3B). This correlated with a significant increased survival advantage for the RAD51 depleted cohort (p=0.025)(Figure 3C).

### Loss of RAD51 inhibits metastatic seeding of xenografts *in-vivo*

**Figure 4:**
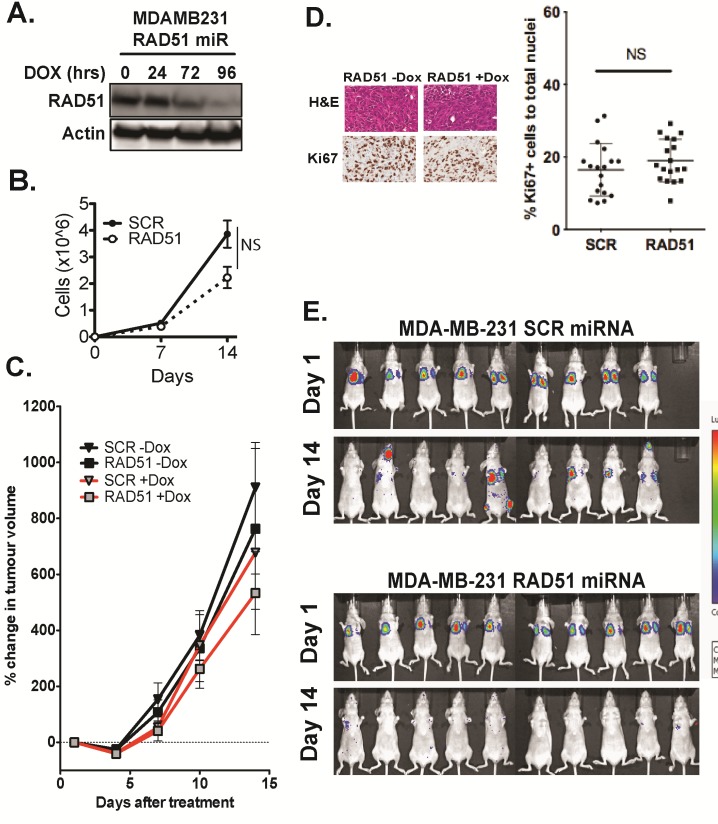
Depletion of RAD51 inhibits metastatic seeding of the lungs in-vivo (A) Western blot analysis of knockdown of RAD51 protein expression using an inducible RAD51mir construct after 96 hours exposure to 5µg/ml Doxycycline. (B) Comparison of growth rate between MDA-MB-231 cells depleted of RAD51 and scrambled controls revealed no significantly affect in proliferation in vitro over 2 weeks (p=0.171) +/− SEM. (C) Primary tumour volume was monitored by changes in luciferase signal in cohorts of 10 mice over 2 weeks. Knockdown of RAD51 did not affect primary tumour growth of MDA-MB-231 cells transplanted into the mammary fat pad % tumour volume change +/−SD. (D) Histological analysis of mammary tumours show that knockdown of RAD51 did not affect growth rate in vivo as measured by Ki67. Results are quantification of 5 fields of 2 tumours of each cohort, +/− SEM (p=0.260). Images are 100X magnification. (E) Analysis of xenograft seeding and potential metastatic dissemination of MDA-MB-231 cells show that 1×10^6^ doxycycline induced RAD51 depleted cells injected intravenously were able to seed the lungs initially but after 14 days were unable dissemination and form metastases.

In order to further study the metastasis-inhibitory effect of Rad51 in human breast cancer cells, MDA-MB-231 cells harbouring a stable integration of the inducible shRAD51 were injected into separate cohorts of immunocompromised mice by orthotopic implantation and via tail vein injection after inducing knockdown of RAD51 for 96-hours with doxycycline (4 µg/ml) (Figure 4A). Growth of the primary tumor and seeding of secondary sites was monitored by bioluminescence imaging. Depletion of RAD51 expression did not significantly reduce proliferation rates of the MDA-MB-231 cells in vitro (p=0.221) (Figure 4B) and primary tumour growth was not affected in vivo (Figure 4C), with no significant difference in proliferation as measured by immunostaining of Ki67 (Figure 4D). In the tail vein injection group, successful implantation of RAD51 competent and depleted cells in lungs was recorded on day 1, yet after 14 days none of the mice transplanted with RAD51 depleted cells displayed colonization in either the brain, bone or lung compared to 56% of the mice in the control group (Figure 4E). Of note after a further 14 days (day 28) only 50% of mice transplanted with RAD51 depleted cells developed metastases compared with 89% of the control mice. To gain mechanistic insights on the role of RAD51 in promoting the colonization of secondary sites, we focused on phenotypic changes induced by knockdown of RAD51 in MDA-MB-231 cells. Metastatic colonization of basal breast cancers can require reversion from a mesenchymal to a more epithelial like phenotype (MET transformation) and change in stem-ness promoting migration and survival. After 7 days of knock-down of RAD51 in MDA-MB-231 cells, we observed a change in morphology in a sub-population of cells consistent with a more epithelial, flattened shape and possible MET via actin cytoskeletal rearrangement (Supplementary Figure 3A). However there was no corresponding change in expression of stem cell markers CD24/CD44 (Supplementary Figure 3B) and vimentin or E-cadherin at the cell-cell junction (Supplementary Figure 3C) or doubling rate (Supplementary Figure 3D). Taken together these results suggest that RAD51 depletion is able to delay colonization/ seeding at secondary sites, via a mechanism that alters actin cytoskeleton with negligible impact on mesenchymal phenotype/ stemness of the cells.

**Figure 5:**
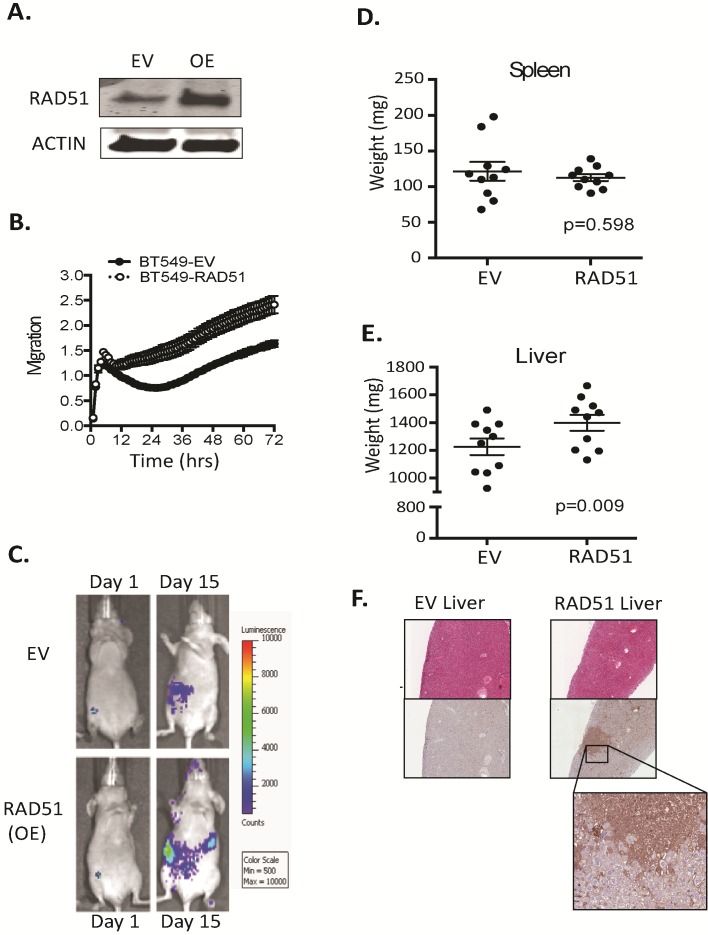
RAD51 can transform poorly metastatic cells (A) Western blot analysis performed on human BT549 engineered for overexpression of RAD51. (B) Analysis of the migration potential of the overexpressing line and control lines was examined over 72 hours. (C) Cohorts of 10, 5 week-old nude (NU-*Foxn1nu*) mice were injected into the mammary fat pad with poorly metastatic BT549 line overexpressing RAD51 or a control empty vector. Tumour cell dissemination was monitored by luciferase expression. (D vs E) Mice harbouring overexpressing tumour cells displayed increased liver weight but not spleen weight +/−SEM. (F) Metastatic burden was confirmed by vimentin positive colonies in the liver.

To confirm a role for RAD51 in breast cancer metastasis in vivo, BT549 cells that had been engineered to express firefly luciferase were transfected with RAD51 expression plasmid or empty vector (EV) (Figure 5A). The gain of function increased the migration rate of the cell line as measured by transwell assays (Figure 5B). Next we assessed the degree of spontaneous liver metastasis by orthotopic implantation. As previously observed, BT549 established low level of spontaneous metastases in the liver [19] and enhanced RAD51 expression resulted in a significant increase in metastatic burden in the liver compared to the control cohort (EV) (Figure 5C). This was not observed in the spleen (Figure 5D vs 5E). Each cohort of 10 mice was examined and 1 mouse in the control cohort had vimentin positive macro-metastases while 6 mice in the Rad51 overexpressing cohort had vimentin positive macro-metastases (Figure 5F). Taken together these results indicate that RAD51 depletion suppressed the metastatic spread of MDA-MB-231 cells and RAD51 overexpression enhanced the metastatic spread of BT549 cells, suggesting that RAD51 increases the metastatic potential of breast cancer *in vivo*.

### RAD51 regulates metastatic gene expression as a possible transcriptional co-factor

The change in cell morphology of sub-population of RAD51-depleted cells, motivated us to determine whether RAD51 expression level affects metastasis via changes in both migration potential and gene expression; including cell adhesion, extracellular matrix, proliferation and/or transcription factor genes. Knockdown of RAD51 in MDA-MB-231 cells impeded migration (Supplementary Figure 3A), while overexpression in Hs578t cells enhanced migration (Supplementary Figure 3B). We also observed increased expression of c/EBPβ in both Hs578t and BT549 cells overexpressing RAD51 (Supplementary Figure 4). Next we performed a metastasis specific gene expression array on the same cell lines. A total of 82 genes previously implicated in processes relevant to tumor progression were screened, of which 25 were selectively induced with overexpression of RAD51 and repressed with knockdown of RAD51 (Supplementary Fig 5C). In particular, reduced RAD51 expression correlated with increased expression of genes that inhibit metastatic invasion, including; *CCL7, BRMS1, NME4* and *TIMP3*, and a corresponding decrease in expression of genes promoting metastatic invasion and proliferation; *MMP13, MMP11, MMP10, MMP3, MMP7, MTA1*, IL1β*, TCF20*, and oncogenes *H-RAS, K-RAS, MDM2* and the mutated form of *TP53.* Four (namely, MMP2, MET, NF2 and TP53) of the 25 identified genes are expressed in aggressive lung metastases from breast cancer [20]. We also confirmed upregulation of 8 of the 25 genes in BT549 cell line overexpressing RAD51 that showed enhanced metastatic spread to the liver in mice. We also determined that 5 (*MMP11, MMP13, SMAD2 TGFβ, TP53*) of the 25 are regulated by the transcription factor CCAAT/enhancer binding protein beta (*c/EBPβ*) when further analysed for transcription factor binding motifs using Ingenuity analysis.

### RAD51 is required for transcriptional activity of c/EBPβ

**Figure 6:**
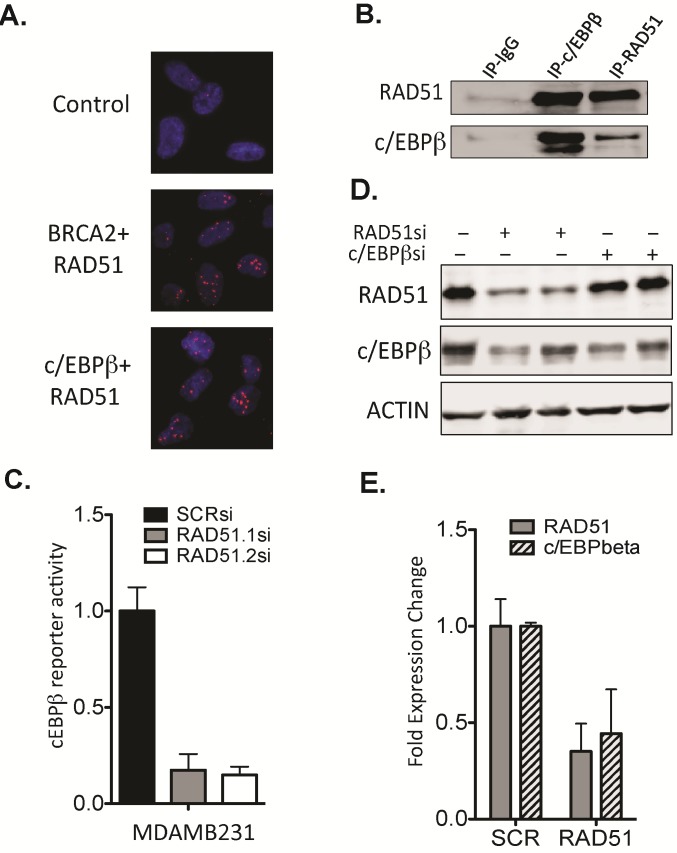
RAD51 forms a complex with the transcription factor c/EBPβ (A) A Duolink™ proximity ligation assay was used to analyse close proximity of RAD51 and c/EBPβ in MDA-MB-231 cells in situ (<40nm). The interaction between RAD51 and c/EBPβ was observed at similar levels as the BRCA2 positive control. (B) Coimmuniprecipitation of endogenous c/EBPβ and RAD51 was performed in MDA-MB-231 cells with recipricol antibodies. (C) A c/EBPβ reporter activity construct was used in MDA-MB-231 to analyse the effect of RAD51 knockdown on c/EBPβ transcription. Using two independent RAD51 targeting RNAi significantly inhibited c/EBPβ transcription (p<0.0001). (D) Western blot analysis of protein expression levels using RNAi directed towards both c/EBP and RAD51 was performed in MDA-MB-231 cells. The knockdown of RAD51 resulted in loss of c/EBPβ expression to comparable levels using targeted siRNA. (E) Analysis of gene expression using qRT-PCR. Knockdown of RAD51 resulted in associated loss of c/EBPβ mRNA expression in MDA-MB-231 cells. Experiments C and E are plotted as average of 3 independent experiments ±SEM.

c/EBPβ is important for mammary gland development and its expression is deregulated in breast cancer [21]. RAD51 has previously been shown to act as a co-factor for c/EBPβ and augment its ability to modulate basal and Tat-induced activation of HIV-1 LTR [22]. We confirm for the first time in breast cancer cells that RAD51 and c/EBPβ interact in situ (Figure 6A) and confirmed this by co-immunoprecipitation of endogenous proteins (Figure 6B). RAD51 expression levels also determines c/EBPβ transcriptional activity with up to 80% reduction in activity observed with depletion of RAD51 using two differently targeting RNAi constructs (p=2.8×10^−9^) (Fig 6C). To confirm RAD51 associated changes in expression of c/EBPβ target genes we performed chromatin immunoprecipitation (ChIP) of c/EBPβ at the target genes identified in array in MDA-MB-231 cells with or without knockdown of RAD51. After depletion of RAD51 there was a reduction in the enrichment of c/EBPβ at the promoter of MMP11, MMP13, TGFβ and SMAD2 (Supplementary Figure 6). Interestingly, targeting of RAD51 by two different siRNAs directed against different regions of RAD51 lead to an associated downregulation of c/EBPβ protein (Fig 6D). We also observed co-regulation of expression with some reduced mRNA expression of c/EBPβ after depletion of RAD51 (Fig 6E). This suggests that RAD51 and c/EBPβ exist as a part of complex wherein RAD51 likely controls c/EBPβ transcription affecting metastatic gene expression levels and the pro-metastatic cell phenotype profile. The molecular mechanism by which this occurs represents a new function for a DNA damage response protein and is part of ongoing research.

## DISCUSSION

Although DNA repair proteins are considered fundamental to the development of breast cancer, their contribution in pathogenesis of sporadic breast cancer remains an understudied area. Using cell line, immunohistochemistry, in silico and animal model analysis we find that high RAD51 expression correlates with aggressive breast cancer and its loss retards metastasis. We also observed high RAD51 expression in conjunction with p53 mutation or loss. This provides an additional driver of mutation and a likely mechanism for overexpression via loss of p53-mediated repression of RAD51 at the RAD51 promoter [9, 23]. RAD51 also contains FoxM1 binding sites in its promoter and its expression can be regulated in glioblastoma by this transcription factor [24]. Although RAD51 also contains promoter-binding sites for c/EBPβ, it is currently not known to regulate RAD51 expression in the cancer setting. Interestingly c/EBPβ can repress p53 expression [25] suggesting a possible mechanism for RAD51 overexpression. Thus, the mechanism of increased transcriptional upregulation of RAD51 in cancers has not been fully defined but it is unlikely to involve gene amplifications or fusions [26]. Using breast tissues TMA, we confirmed that high level of RAD51 correlates with advanced histological grading in breast cancer, consistent with a previous report [3].. Notably, RAD51 is highly expressed in matched lymph node and brain metastasis compared to primary tumor. Overall, these data indicate that RAD51 overexpression might have significant impact on progression and metastasis of breast cancer.

Overexpresison of DNA damage response genes are known to support cancer [9]. However DNA damage proteins have not previously been strongly linked with metastatic progression. We show that RAD51 is crucial not only for primary tumor growth but also metastatic spread of 4T1.2 cells. Using tail vein assay of cancer metastasis in MDA-MB231 cells, we show that RAD51 dramatically promotes appearance of lung metastasis. Notably, these results cannot be explained by effect of RAD51 on cell growth, as RAD51 depletion did not effect cell proliferation and doubling time in vitro and primary tumor growth in vivo in MDA-MB231 xenografts. Strikingly, overexpression of RAD51 in BT549 cells significantly increased spontaneous metastasis to liver. The data presented here constitute the first direct evidence of the role of RAD51 promoting or enhancing metastastic progression.

Consistent with changes in metastatic potential, we found that RAD51 increases the expression of prometastatic genes and reduces the expression of metastasis suppressor genes. Amongst these were several c/EBPβ target genes. We further demonstrated that RAD51 and c/EBPβ exist as part of a complex wherein RAD51 controls c/EBPβ transcription. Consistent with this c/EBPβ binding at its target genes and transactivation of c/EBPβ reporter was reduced in RAD51 depleted cells. Our array also defined target genes of c/EBPβ (regulated by RAD51 expression) that are required for invasion. Stromal cells largely express MMPs, however we find distinct changes in their expression in breast cancer cell lines. It is suggested that cancer cell lines that have already undergone epithelial-mesenchymal transition, such as triple negative breast cancer cells up regulate MMPs as part of promoting cell motility, leading to migration from the primary tumour and intravasation [27]. This has also been observed in xenografts of human breast cancer cell lines [28]. We find that both MMP11 and MMP13 levels are regulated by RAD51 expression. MMP13 is upregulated in metastatic breast cancer patient samples [29] and is part of a gene signature that is a predictor of prognosis in breast cancer lymph node metastasis [30].

Thus it appears that RAD51 has two distinct roles in promoting metastasis; the first is based on the increased repair and stabilizing the cancer genome allowing spread of tumor cells [8, 9] and the second as a regulator of c/EBPβ in promoting pro-metastatic gene expression. The stabilization of the cancer genome means that deregulated RAD51 is promoting aberrant DNA repair that allows advantageous mutations beyond those required for primary tumors. For example ectopic overexpression of RAD51 protein in mouse embryonic stem cells leads to a novel spectrum of recombination events, including multiple chromosome rearrangements and aneuploidy [7]. The second role regulates metastasis, via modulation of gene expression as a cofactor for c/EBPβ. c/EBPβ transcriptional activity is determined by the levels of isoforms LAP1, LAP2 and LIP. LIP forms suppressive heterodimers with the other isoforms. c/EBPβ has been associated with hematopoietic and adipocyte differentiation [31, 32] and implicated as an essential mediator of mammary gland development[33]. Therefore we can assume that c/EBPβ is able to regulate gene expression in breast tissue resulting in growth and differentiation. Indeed in the breast cancer setting, deregulation of LIP function contributes to escape of cytostatic effects of TGFβ and contributes to tumour metastasis [33-36]. We observe that RAD51 is strongly associated with breast cancer pathology progression, high-grade tumours and metastasis. The combination of RAD51 contributing to enhancing c/EBPβ expression efficiency is undeniably a potent combination in driving metastatic progression. Supporting this hypothesis is the number of oncogenes and tumour suppressors (*H-RAS, KRAS, c-MYC, TP53*) revealed in our array that are regulated in response to RAD51 expression levels.

The role of transcriptional co-factor, a regulator of metastatic gene profiles makes RAD51 a possible biomarker and new therapeutic target. RAD51 has been suggested as possible biomarker to predict sensitivity to PARP1 inhibitor treatment and potential predictor for metastatic survival [37, 38]. We believe our findings have clinical implications for treating aggressive metastatic triple negative breast cancer and other cancers that have acquired overexpression of RAD51 and is the focus of ongoing work.

## MATERIALS AND METHODS

### Clinical samples

Formalin-fixed paraffin embedded (FFPE) tumor blocks were retrieved from the archives of the Royal Brisbane and Women's Hospital (Australia), Sullivan and Nicholades Pathology (Australia), various sources outlined previously[39] and the Kathleen Cuningham Foundation Consortium for research into Familial Breast Cancer (kConFab, Australia). There were several sets of samples analyzed, outlined in Table 1, including primary breast cancers and metastases on tissue microarrays (TMA): sporadic primary tumors (n=235), familial primary tumors (n=560), lymph node (LN) metastases (n=28), matched primary and brain metastases (n=39). For these cases the immunohistochemistry data for ER, PR and HER2 were available. We also analyzed 23 cases on whole sections, in which multiple stages of progression were analyzed: in situ carcinoma, invasive carcinoma, LN metastasis. The use of tumor samples was approved by the local research ethics committees.

### Immunohistochemistry

Immunohistochemistry (IHC) was performed on TMA sections, whole tissue sections and resected murine tumors to evaluate RAD51 protein expression in breast cancer. Sections were cut at 4 µm, mounted on silane-coated slides and subjected to antigenic retrieval in EDTA [pH 8.0] for 2 minutes at 105°C. IHC was done using the anti-RAD51 primary antibody (SC 8349, Santa Cruz Biotech; 1:40 dilution, 2 hours incubation at 37C) and the MACH 2™ Polymer Detection system according to the manufacturer's (Biocare Medical, Concord, CA, USA). Staining was visualized with 3,3'-Diaminobenzidine (DAB) and a haematoxylin counterstain. Germinal centre lymphocytes in normal lymph nodes served as a positive staining control for RAD51 due to the high rate of DNA repair associated immunoglobulin expression rearrangements. The staining was assessed semi-quantitatively by analysis of RAD51 positive cells with nuclear staining of RAD51 rather than cytoplasmic considered positive, with additional arbitrary thresholds of intensity of staining, compared to positive controls.

### Cell culture, transfections and generation of inducible RAD51 knockdown and overexpression of RAD51 in cells

Breast cancer cell lines MDA-MB-231, BT549, Hs578t and the murine 4T1.2 were grown in DMEM media supplemented with 10% foetal bovine serum. All cell lines except 4T1.2 identity was confirmed by short tandem repeat (STR) profiling. Stealth siRNA (RAD51.1 and RAD51.2) and control siRNA oligos were purchased from Invitrogen. siRNAs were transfected using Lipofectamine 2000 (Invitrogen) as per manufacturer's instructions and samples were analyzed 48 hrs after transfection. Stable inducible knockdown cells were generated by integration of RAD51-mir (5'-CTCGGTACTGTGTTGTTCGTTA-3') and scrambled-mir (5'-GTGTAACACGTCTATACGCCCA-3') constructs into TMP-IRES-GFP vector and transfected into MDA-MB-231 and 4T1.2 cell as described previously by Dickins et al.[40]. Overexpression was performed using Origene precision shuttle mammalian vector with full length human RAD51 and c-terminal tGFP tag and transfected using Lipofectamine 2000 (Invitrogen) as per manufacturer's instructions. Cells were purified based on GFP expression.

### Immunoblotting

Protein lysates were prepared via whole cell lysis in ice-cold lysis buffer (150mM NaCl, 10mM Tris-Cl pH 7.4, 5mM EDTA, 1% Triton X-100) supplemented with protease inhibitors (Leupeptin, Pepstatin and PMSF, Sigma Aldridge). Immunoblots were probed with anti-RAD51 (Santa Cruz Biotech), anti-PARP (Millipore), anti-PAR (BD Biosceinces), DNA-PK;P2609 (Cell Signaling) and anti-53BP1 (Bethyl), and with anti-Tubulin (Sigma) and anti-ß-Actin, (Sigma) as controls. Membranes were developed using fluorescent labeled secondary antibodies and visualized using the Odyssey system. Protein expression levels were determined by optical density versus actin loading controls using Image J software (NIH).

### *In-vivo* studies

Cohorts of 5 week old female nude NU-*Foxn1nu* or Balb/c mice established 40mm^3^ inguinal mammary fat pad tumors from 5×10^6^ cells of MDA-MB-231/BT549 or 1×10^6^ cells of 4T1.2 into the left hind flank. Tumour were either retained or resected and the mice placed on a doxycycline water 4 µg/ml to induce knockdown. Non-induced and empty vector (EV) cohorts served as controls. Tumor growth was monitored by caliper measurement and visualized using luciferase-mediated live animal imaging with the IVIS100 Xenogen system (Caliper Life Sciences).

### *In-vivo* metastatic seeding

Female nude mice NU-*Foxn1nu* (5 weeks old) were injected intravenously with 1×10^6^ doxycycline induced MDA-MB-231-RAD51mir or MDA-MB-231-SCRmir cells. Seeding of the lungs was visualized using luciferase-mediated live animal imaging and monitored weekly for establishment of tumors. Mice remained on doxycycline water for the duration of the experiment.

### Migration studies

3×10^4^ cells were seeded in Xcelligence 16 well CIM-transwell plate without serum (Roche) and monitored for 20 -36 hours for migration towards a 20% serum gradient.

### Metastatic gene expression Array

RNA was isolated from 5 µg/ml doxycycline induced and non-induced MDA-MB-231-RAD51mir or MDA-MB-231-SCRmir cells and Hs578T-EV (empty vector) and Hs578T-RAD51GFP using QIAGEN RNAEasy Mini Kit (Cat 74104). cDNA was synthesized using *RT^2^ First Strand Kit* (QIAGEN Cat 330401). cDNA was loaded in duplicate on The Human Tumor Metastasis *RT^2^ Profiler*™ PCR Array containing 82 genes (QIAGEN-Cat PAHS-028G-4) and analyzed on Roche Lightcycler 480. Fold expression change was calculated against actin and expressed as base-two exponential increase in RNA levels (2ΔΔCt)+/− SEM.

### Statistical methods

Statistical assessment of immunohistochemistry data was performed in GraphPad Prism V5.0c using Chi squared test with Yates correction (95% confidence interval) and Fisher's exact test to compare RAD51 status between defined subgroups of samples. Significance of biological experiments was assessed with student t-tests (two tailed) +/−SEM.

## SUPPLEMENTARY FIGURES AND TABLES


