# Supplementary material for: Rad51 supports triple negative breast cancer metastasis
Source: Oncotarget. 2014 Apr 27;5(10):3261–72. doi: 10.18632/oncotarget.1923 (PMC4102808; doi:10.18632/oncotarget.1923)
Supplement: Supplementary file 1 [file oncotarget-05-3261-s001.pdf]

Rad51 supports triple negative breast cancer metastasis.

Supplemental Material

Supplementary Table 1: RAD51 expression metabric-analysis.

Comparison	RAD51 Low	RAD51 high	Significance
**Age (years)**			
<50	180 (42%)	246 (58)	p=1.56E-02
50-74	614 (48%)	655 (52%)	n.s.
75-100	151 (51%)	146 (49%)	n.s.
**Tumour size**			
<2cm	320 (50%)	316 (50%)	n.s.
>2cm <5cm	555 (46%)	639 (54%)	n.s.
>5cm	71 (47%)	81 (53%)	n.s.
**Tumor Grade**			
Grade 1	61 (69%)	28 (31%)	p=5.0E-6
Grade 2	315 (55%)	261 (45%)	n.s.
Grade 3	304 (36%)	533 (64%)	p<1.0E-6
**Ki67 expression**			
Low	673 (63%)	395 (37%)	
High	404 (39%)	644 (61%)	p<1.0E-6
**Histological subtypes**			
ER negative	137 (31%)	303 (69%)	p<1.0E-6
ER positive	783 (52%)	725 (48%)	
PR negative	377 (40%)	566 (60%)	p<1.0E-6
PR positive	568 (54%)	481 (46%)	
HER2 negative	791 (51%)	755 (49%)	
HER2 positive	152 (34%)	289 (66%)	p<1.0E-6
- non TNBC	866 (50%)	859 (50%)	
Triple negative (TNBC)	68 (27%)	182 (73%)	p<1.0E-6
**Intrinsic subtypes**			
Luminal A	482 (67%)	239 (33%)	p<1.0E-6
Luminal B	131 (27%)	361 (73%)	p<1.0E-6
HER2-enriched	98 (41%)	142 (59%)	p=2.9E-02
Normal-like	149 (74%)	53 (26%)	p<1.0E-6
Basal-like	82 (25%)	249 (75%)	p<1.0E-6
**TP53 mutation**			
Wildtype	228 (41%)	329 (59%)	
Mutatnt	20 (24%)	64 (76%)	p=2.67E-03
**Survival**			
Overall survival			
Alive	508 (47%)	573 (53%)	
Dead	428 (48%)	463 (52%)	n.s.
Alive at 1 year	680 (46%)	810 (54%)	
Dead at 1 year	9 (31%)	20 (69%)	n.s.
Alive at 3 years	612 (37%)	677 (53%)	
Dead at 3 years	57 (30%)	133 (70%)	p=6.0E-6
Alive at 5 years	506 (49%)	531 (51%)	
Dead at 5 years	121 (34%)	231 (66%)	p=3.0E-6

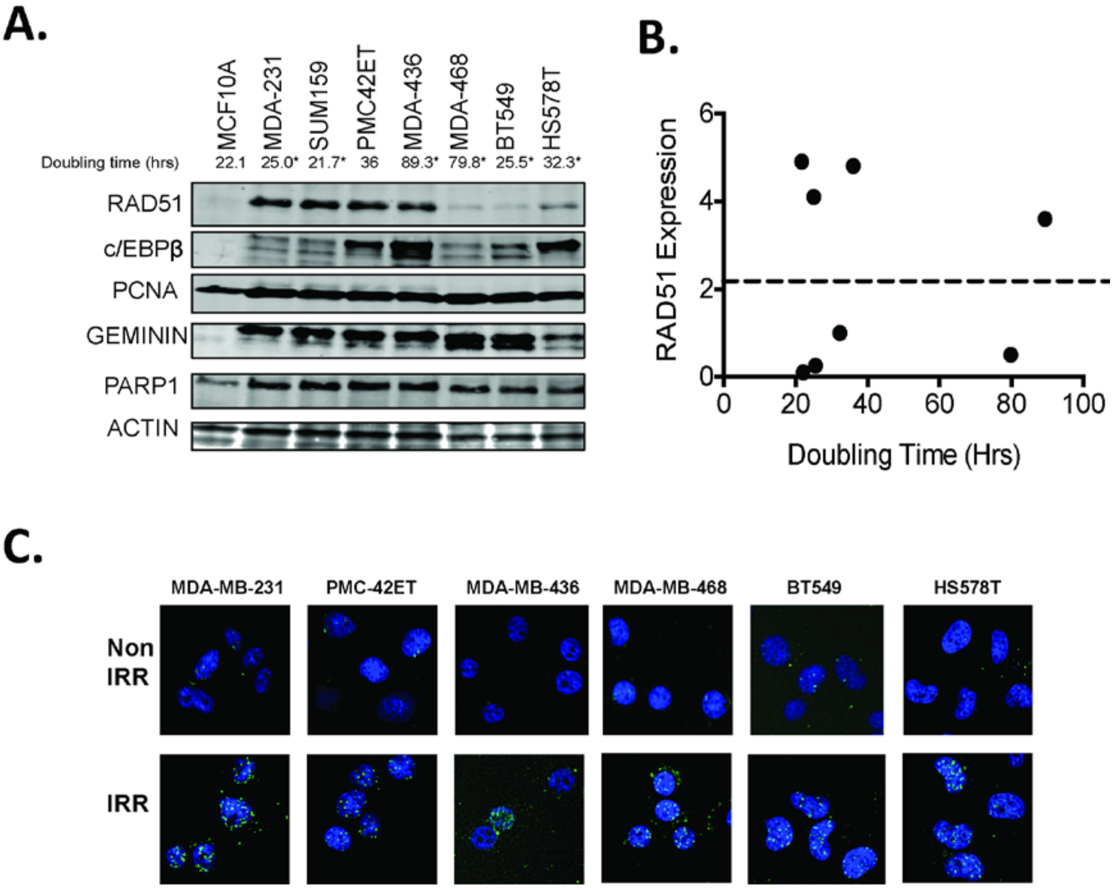

Supplementary Figure 1: RAD51 expression does not correlate with growth rate or function.

(**A**) Expression of RAD51, c/EBPβ, PCNA, Geminin, PARP1 was analysed in 7 basal breast cancer cell lines and compared to MCF10A cell line (near normal mammary epithelial cell line derived from a patient with benign fibrosarcoma) with actin serving as a loading control. Doubling rates of the cell lines with asterix (*) were adapted from Daemen et al, 2012 Breast Cancer Res Treat. Sep;135(2):505-17 Table 1. (**B**) Doubling rates versus RAD51 expression levels calculated as a ratio to actin, display no correlation with a coefficient of R^2=0.0012. (**C**) All cell lines utilized in this study have functional RAD51 with foci formation in response to 6 Gy irradiation after 6 hours.

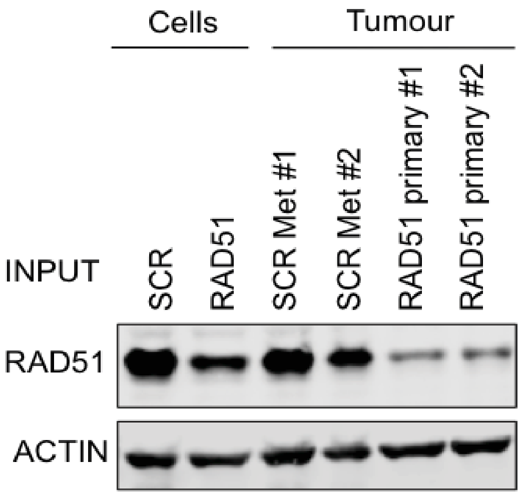

Supplementary Figure 2: RAD51 overexpression is observed in mouse metastatic tumours. Western blot analysis of RAD51 expression from 2 primary tumours from RAD51 knockdown induced mice and 2 metastatic tumours from control scrambled induced mice after 28 days. The RAD51 knockdown was sustained at the primary site in contrast to overexpression of RAD51 observed in the metastatic samples.

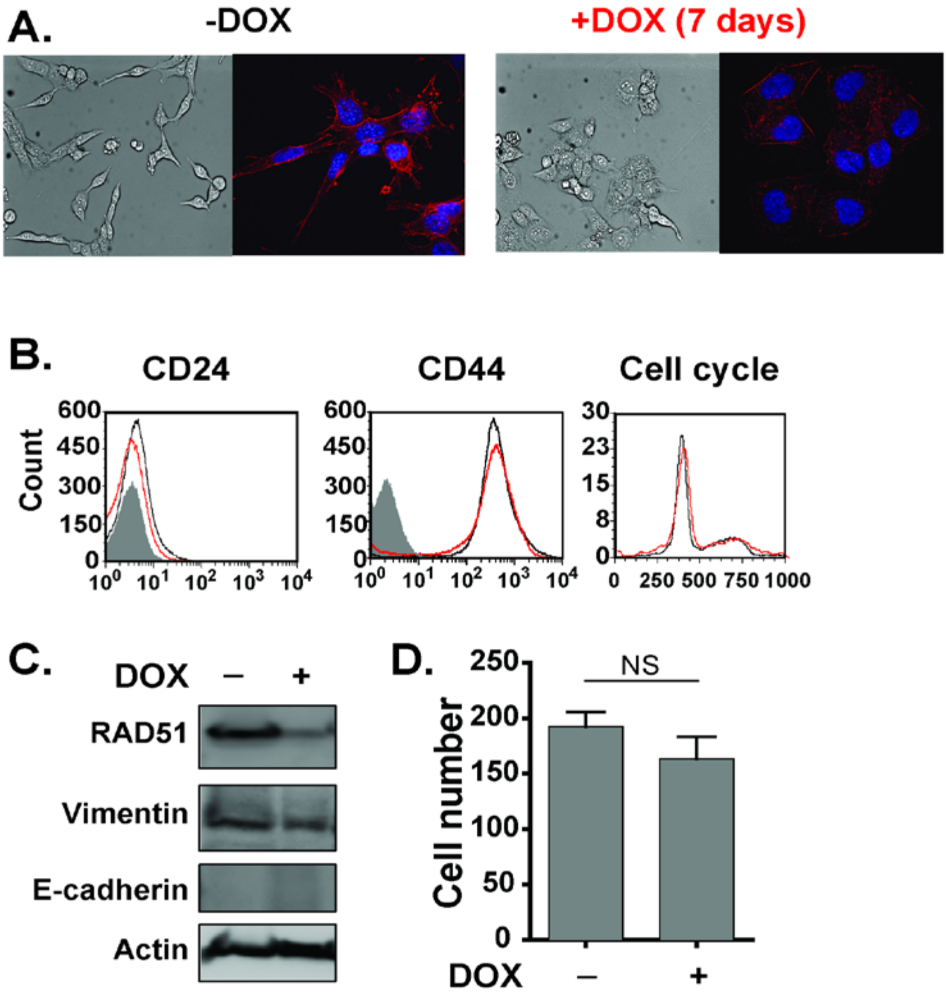

Supplementary Figure 3: Knockdown of RAD51 does not affect stemness or cell growth. MDAMB231-RAD51mir cells were incubated with and without 5µg/ml Doxycycline for 7 days and analyzed for, (**A**) morphological changes, bright field in the left panels and phalloidin/DAPI fluorescence staining in the right panels. (**B**) FACS analysis for stem cell markers CD44, CD24 and cell cycle (red line represents knockdown and shaded plot represents isotype control), (**C**) Western blot analysis of protein expression of vimentin and E-cadherin and (**D**) cell doubling rate. Figure D represents the average +/-SEM of 5 independent experiments.

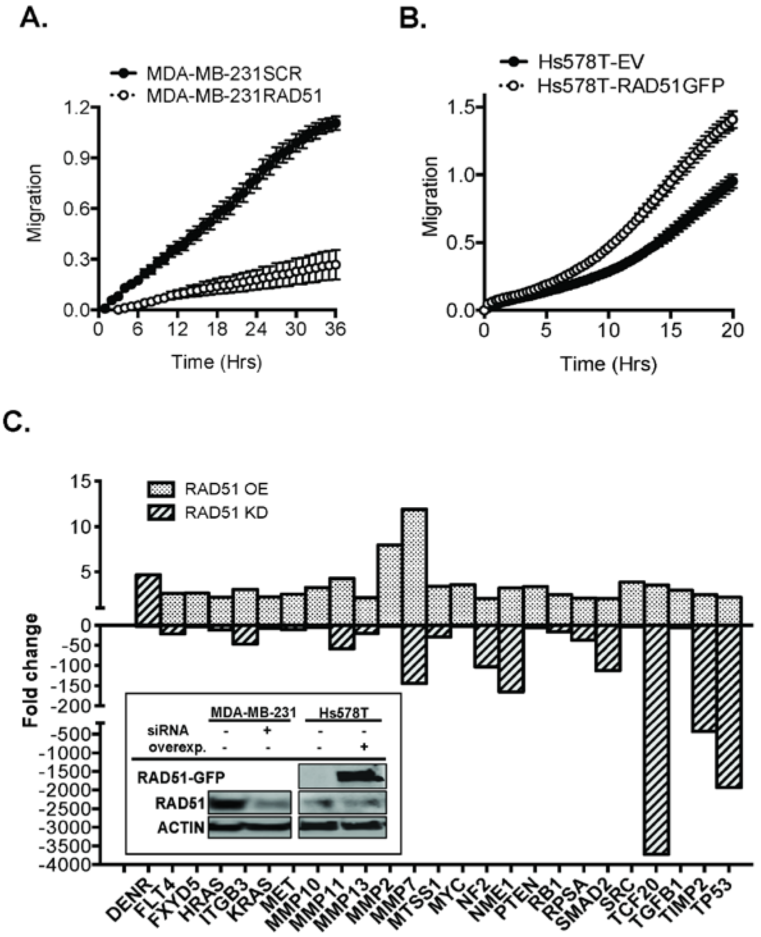

Supplementary Figure 4: RAD51 expression levels affects metastatic gene expression.

The migration potential of 2 different basal breast cancer cell lines was analysed. (**A**) MDA-MB-231 cells depeletd of RAD51 displayed significant reduction in migration compared to the scrambled control line over 36 hours ($p<0.0001$) and (**B**) Hs578T RAD51 overexpressing displayed significant enhancement in migration compared to the empty vector control line after 20 hours ($p<0.005$). (**C**) Fold change in expression of 82 metastatic related genes were compared between MDA-MB-231 cells with and without RNAi induced knockdown of RAD51 and Hs578T cells expressing low baseline levels and induced overexpression of RAD51. 25 genes demonstrated coordination induction and repression in response to changes in RAD51 expression levels. Represented data is average of duplicate experiments. (**Insert**) Western blotting confirmed RAD51 status of the cell lines.

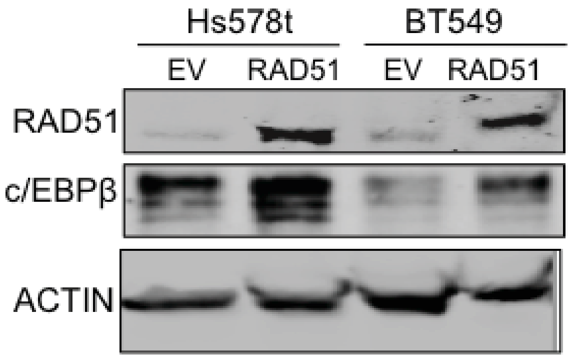

Supplementary Figure 5: Analysis of RAD51 overexpression. Overexpression of RAD51 correlates with increased expression of c/EBPβ in both Hs578t and BT549 breast cancer cell lines.

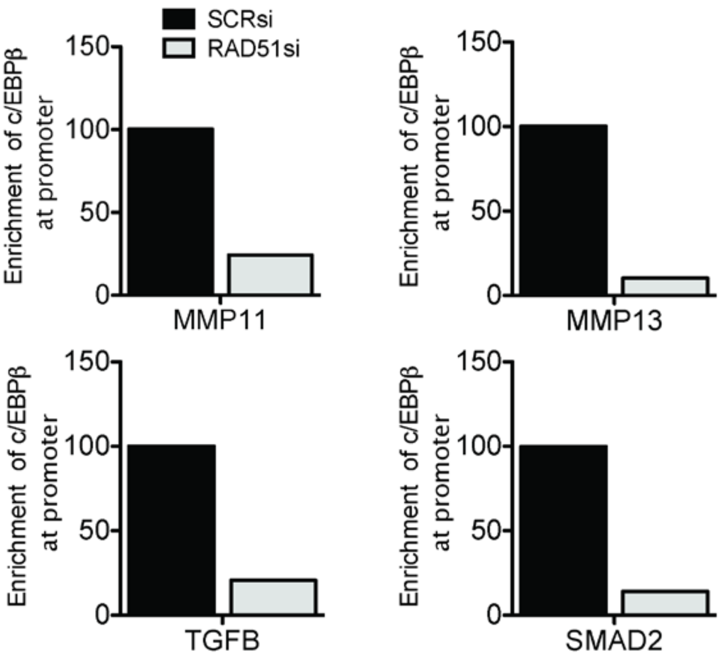

Supplementary Figure 6: Depletion of RAD51 retards c/EBPβ transcriptional activity.

Analysis of c⁄EBPβ binding at the promoter regions of its target genes was performed using chromatin-immunoprecipitation. Induced depletion of RAD51 resulted in reduced binding of c⁄EBPβ at the promoter of MMP11, MMP13, TGFβ and SMAD2 .
